# Horseshoe Kidney Injury Managed With Percutaneous Nephrostomy for Urinoma: A Case Report

**DOI:** 10.7759/cureus.86306

**Published:** 2025-06-18

**Authors:** Ryota Kiyohara, Daisuke Mizu

**Affiliations:** 1 Emergency Medicine, Osaka Red Cross Hospital, Osaka, JPN

**Keywords:** horseshoe kidney, nephrostomy, renal injury, trauma, urinoma

## Abstract

Horseshoe kidney injuries are rare, and whether their management is similar to that of common renal injuries remains unclear. We present the case of a 41-year-old man who bruised his abdomen while riding a bicycle and experienced persistent abdominal pain. His vital signs were stable, but he had significant tenderness below the umbilicus. Contrast-enhanced abdominal computed tomography revealed a horseshoe kidney injury, urine leakage, and a urinoma. Conservative treatment was performed, but the urinoma continued to enlarge. Percutaneous drainage was performed on the seventh day in the hospital. The urinoma improved, and the patient was discharged on day 24. As in this case, horseshoe kidney injuries can easily lead to deep damage and associated complications compared to a normal kidney. Therefore, they are more likely to require surgical intervention. Although there are many methods of intervention, including endovascular treatment for bleeding, surgery, and drainage for urinoma, the management of horseshoe kidney injury requires consideration of the complex anatomy of the vascular and urogenital systems and identification of appropriate treatment modalities.

## Introduction

Traumatic kidney injury occurs in approximately two cases per 100,000 people in Japan [1]. The majority result from blunt trauma [1-3], with >90% of blunt kidney injuries being managed conservatively [2]. In addition, complications such as urine leakage, urinoma, and renal abscess occur in approximately 5.2% of cases [4]. A urinoma is a cyst formed by encapsulation of leaked urine due to trauma or other causes. Urinomas often resolve spontaneously; however, sometimes they may require percutaneous drainage or ureteral stenting. Minimally invasive treatments, such as endovascular treatment for bleeding and urine leakage drainage, are required in approximately 2% of cases, whereas open surgery is required in approximately 1% of cases [5], suggesting that traumatic kidney injury is rarely fatal. However, few reports of kidney injury in morphologically abnormal kidneys exist. An estimated 7-19% of blunt kidney injuries occur in abnormal kidneys [6], and whether the management of these injuries is similar to that of normal kidney injuries remains unclear. Horseshoe kidney is the most common congenital renal anomaly, occurring in approximately one out of 400 individuals [6]. The condition is characterized by the fusion of the two lower poles of the kidneys during their rotation around the main axis. This fusion typically occurs at the narrow region of the renal parenchyma and is often accompanied by anatomic abnormalities in the vascular and urinary systems. Although previous studies of horseshoe kidney injury have reported interventions for hemorrhage [6,7], few studies have reported urinary tract complications, and thus their management remains unclear. We herein report a case of traumatic horseshoe kidney injury caused by abdominal trauma due to a fall, which was successfully managed with nonoperative and percutaneous drainage for a urinoma complication.

## Case presentation

**Figure 1:**
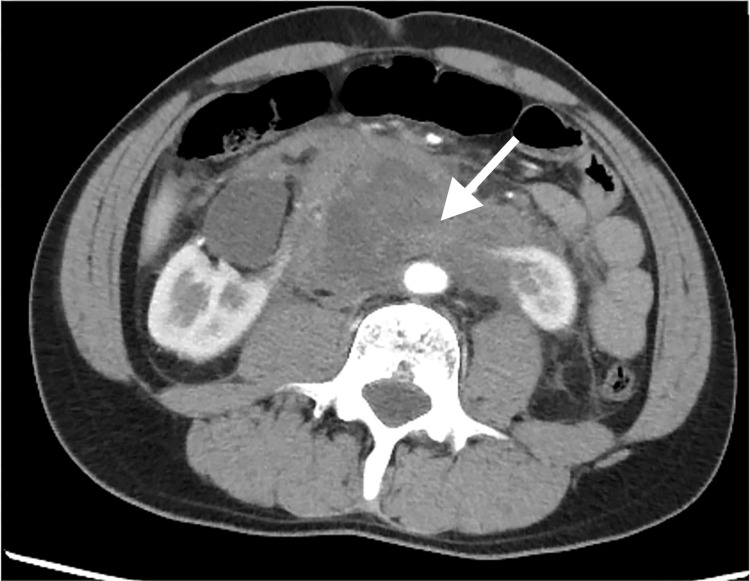
Contrast-enhanced abdominal computed tomography (CT) performed in the emergency department revealed an injury to the isthmus of the horseshoe kidney (arrow).

A 41-year-old man with tetralogy of Fallot and a horseshoe kidney fell while riding his bicycle and hit his lower abdomen against the saddle. The patient experienced persistent abdominal pain and was transported to the emergency department four days after the injury. His vital signs were Glasgow Coma Scale of 15 (E4V5M6), blood pressure of 123/91 mmHg, heart rate of 108/min, respiratory rate of 20/min, oxygen saturation of 95% (room air), and body temperature of 37.1°C Additionally, the patient had abdominal distension, lower abdominal tenderness, rebound tenderness, and bilateral costovertebral angle-tapping pain. Contrast-enhanced abdominal computed tomography (CT) revealed a horseshoe kidney with injury to the isthmus and a retroperitoneal hematoma (Figure 1). No extravasation of the contrast was detected.

**Figure 2:**
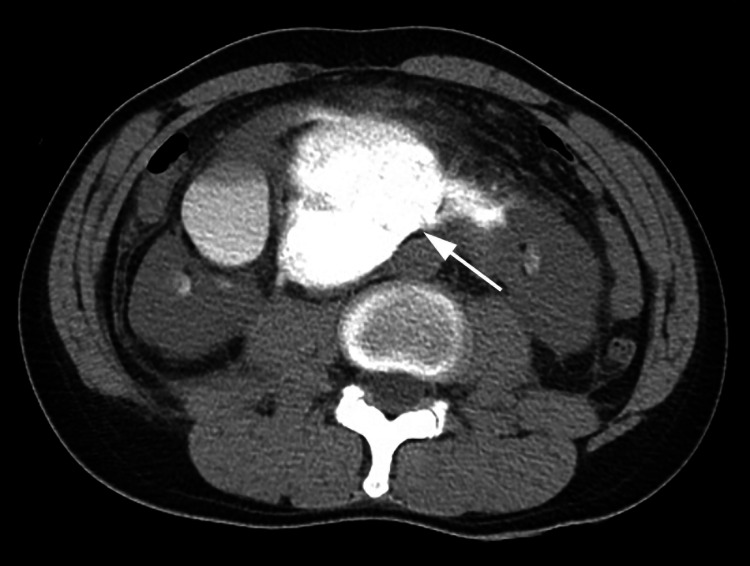
Plain abdominal CT on day 2 revealed contrast accumulation in the retroperitoneum (arrow).

Systolic blood pressure >120 mmHg and heart rate <100/min were maintained during treatment in the emergency department. Vital signs were unremarkable, and no active bleeding was present; therefore, conservative management was decided. Plain abdominal CT on day 2 revealed contrast accumulation in the retroperitoneum, which was initially suspected to be a retroperitoneal hematoma but was later determined to be predominantly attributed to a urinoma (>4 cm) caused by urine leakage (Figure 2).

**Figure 3:**
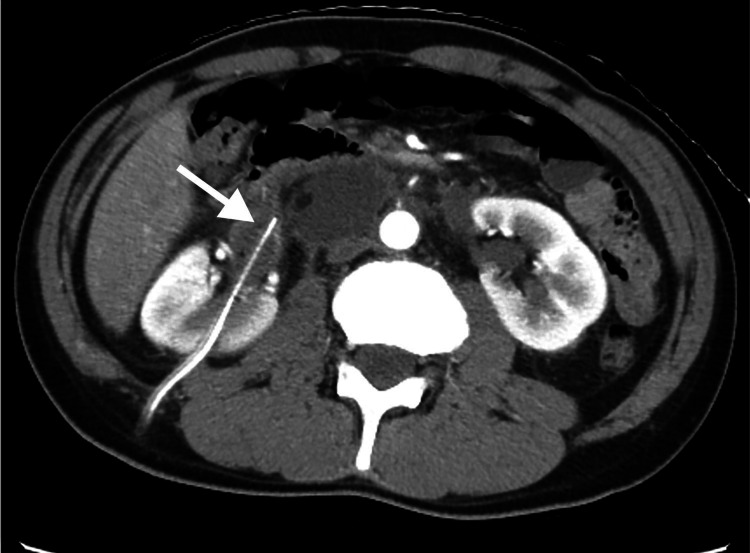
Abdominal CT on day 7 demonstrates a percutaneous nephrostomy catheter placed in the right renal pelvis (arrow).

Percutaneous drainage for the urinoma was performed on day 7 due to fever, elevated inflammatory response on blood tests (white blood cell count: 14,930/μL (normal range: 3300-8600 /μL), C-reactive protein level: 19.9 mg/dL (normal range: 0.0-0.5 mg/dL)), and worsening lower back pain. The left ureter was identified from the kidney to the bladder on CT, but the right ureter was difficult to identify midway. Therefore, drainage was performed on the right renal pelvis, considering the possibility of urine leakage from the right ureter (Figure 3).

After surgery, the patient showed improvement in lower back pain and in the inflammatory response on blood tests. CT on day 15 revealed a reduction in the urinoma size. The patient was discharged on day 24 of hospitalization. The patient was followed up by a urologist seven days after discharge. During the course of the illness, no recurrent episodes of fever or abdominal pain occurred.

## Discussion

In the present case, despite the relatively minor trauma, deep renal damage occurred due to the horseshoe kidney, and percutaneous drainage was performed for urine leakage and urinoma. Previous reports have indicated that even minor horseshoe kidney injuries can be severe and require endovascular treatment or surgery [8], which was also observed in the present case. Because the horseshoe kidney isthmus is located anterior to the lumbar vertebrae, the kidney is susceptible to compression injury from even mild blunt abdominal trauma [6]. Approximately 80% of blunt kidney injuries in Japan are classified in grade I-III according to the American Association for the Surgery of Trauma Classification with an Injury Severity Score (ISS) of 16 [3]. However, the present case was classified as grade IV, consistent with previous cases of horseshoe kidney injuries, which were also reported as grade IV injuries [7]. It is important to consider that even minor trauma can result in more severe injury in cases involving a horseshoe kidney.

Blood clots in the renal collecting system and urinomas >4 cm have been reported as predictors of the requirement for urine leakage intervention [9]. In the present case, a urinoma >4 cm was identified from the beginning, so early intervention could have been considered: plain abdominal CT on day 2 revealed contrast accumulation in the retroperitoneum, which was initially suspected to be retroperitoneal hematoma and was later determined to be predominantly attributed to a urinoma caused by urine leakage (Figure 2). It is important to include the excretory phase in CT for accurate diagnosis. In this case, the excretory phase in CT was not performed during the initial assessment, which led to a failure to detect urinary leakage and urinoma, resulting in a delay in therapeutic intervention. Especially in cases where ureteral injury cannot be ruled out, the excretory phase in CT enables identification of ureteral injury and leads to timely treatment. If the patient's hemodynamics are stable, early drainage should be considered when there is a hematoma or urinoma of a certain size, or when signs of infection, such as abdominal pain or fever, are observed.

In the present case, urinoma intervention was performed on day 7 of admission. Initially, we opted for conservative management because of reports of improvement without invasive intervention [2]. Although drainage was performed due to exacerbation of fever and abdominal pain, earlier intervention may have been appropriate considering the possibility of infection. No consensus exists regarding whether ureteral stenting or percutaneous drainage should be prioritized in cases of urine leakage [10,11]. In the present case, considering the possibility of an infected urinoma, percutaneous drainage was performed to ensure drainage, at the discretion of the urologist. Drainage by the physiologically correct excretory route is preferred, so stenting should always be considered. However, ureteral stenting is often difficult due to urinary tract abnormalities in horseshoe kidney injury. Therefore, percutaneous drainage may be considered from the beginning, with the purpose of preventing infection exacerbation [12]. We should carefully consider the possibility of morphological abnormalities associated with the horseshoe kidney and verify whether the procedure is technically feasible.

## Conclusions

Horseshoe kidney injury is rare; however, emergency physicians should be familiar with its characteristics as they may encounter the condition in clinical practice. Although the therapeutic strategies are not significantly different from those for general kidney injury, it is important to consider that even minor trauma can be more severe in horseshoe kidney injuries. When assessing complications with CT, the urinary excretory phase is useful for differentiating between urine leakage and hematoma. When performing surgical intervention, carefully considering the possibility of morphological abnormalities associated with the horseshoe kidney and verifying whether the procedure is technically feasible is necessary.
